# Physical compatibility of Xuebijing injection with 53 intravenous drugs during simulated Y-site administration

**DOI:** 10.1371/journal.pone.0299694

**Published:** 2024-03-22

**Authors:** Tong Tong, Peifang Li, Haiwen Ding, Ying Huang, Sheng Liu

**Affiliations:** 1 Department of Pharmacy, The First Affiliated Hospital of USTC, Division of Life Sciences and Medicine, University of Science and Technology of China, Hefei, Anhui, P.R. China; 2 Anhui Provincial Key Laboratory of Precision Pharmaceutical Preparations and Clinical Pharmacy, Hefei, Anhui, China; University of Veterinary and Animal Sciences, PAKISTAN

## Abstract

**Objective:**

Xuebijing injection (XBJ) is a commonly used herbal medicine injection in China. However, the physical compatibility of XBJ with other intravenous drugs remains unclear. The purpose of this research is to evaluate physical compatibility of Xuebijing injection (XBJ) with 53 intravenous drugs (including 31 Chinese medicine injections and 22 chemicals) during simulated Y-site administration.

**Methods:**

Y-site administration was simulated in vitro by admixing 0.33 ml/ml XBJ with an equal volume of other diluted 53 intravenous drugs, respectively. Physical compatibility including visual inspection, Tyndall beam, particle limits, turbidity, pH, chromacity value, spectroscopic absorption of 550 nm and 420 nm (A_550 nm_ and A_420 nm_) were observed and assessed at 0, 1, 2, and 4 h. Physical compatibility was defined as all solutions with no color changes, no gas evolution, particulate formation and no Tyndall beam within 4 hours, turbidity changes <0.5 nephelometric turbidity unit (NTU) compared to 0 h, particle limits allowed by the Chinese Pharmacopoeia (Ch.P) 2020 edition, pH changes <10% compared to 0, chromacity value changes <200 compared to 0 h, or photometrical changes of A_420 nm_ <0.0400 or A_550 nm_ <0.0100 compared to 0 h.

**Results:**

XBJ was physically incompatible with 27 of the 53 intravenous drugs tested, 26 were compatible with XBJ for 4 h.

**Conclusions:**

XBJ should not be simultaneously co-administered with 27 of the 53 intravenous drugs during simulated Y-site. If coadministration was inevitable, flushing tube with NS or D5W before and after infusion of XBJ was needed. Assessment included visual inspection, Tyndall beam, turbidity measurement, particle counts, pH measurement, chromacity value measurement and absorption of A_550 nm_ were proved to be valid and robust for the quality control of infusion and compatibility of Chinese herbal injection.

## 1. Introduction

Xuebijing injection (XBJ) is a yellow or brownish-yellow clarified liquid mainly comprising of Honghua (Flos Carthami), Chishao (Radix Paeoniae Rubra), Chuanxiong (Rhizoma Chuanxiong), Danshen (Radix Salviae Miltiorrhiae) and Danggui (RadixAngelicae Sinensis) [1], the excipients are glucose, polysorbate 80 and water for injection. It was licensed in 2004 by the National Medical Products Administration (NMPA, China) [2] and approved for warm and heat diseases, such as fever, shortness of breath, palpitation, irritability and other syndrome of stasis and poison, to treat patients suffering from systemic inflammatory response syndrome induced by infection, combining therapy with the treatment of multiple organ dysfunction syndrome in the period of organ dysfunction. It can also treat severe and critical systemic inflammatory response syndrome and/or multiple organ failure of novel coronavirus infection.

As a commonly used herbal medicine injection in China, XBJ has been administered to critically ill patients for over 15 years. Extensive studies and clinical research have revealed its functions in sepsis [2–4]. The injection exhibits protective mechanisms by antagonizing endotoxins and inhibiting the uncontrolled release of inflammatory mediators from endotoxin-stimulated monocytes/macrophages. Additionally, XBJ improves coagulopathy in disseminated intravascular coagulation, an important risk factor for sepsis mortality [5]. In China, over 250,000 patients receive XBJ treatment annually [6], and its route of administration and safety are well-established. The multiple positive clinical effects of XBJ make it an essential drug for critically ill patients. However, managing the coadministration of multiple drugs, especially those requiring prolonged intravenous infusions, along with XBJ can present challenges.

Due to limited independent catheters for central venous catheters [7], infusions should be ceased and the line flushed before administering other drugs [8]. However, frequent stopping and flushing may affect the patient’s fluid balance [9]. Y-site access enables simultaneous intravenous drug coadministration but can lead to physical incompatibilities between drugs. Our main interest lies in the physical compatibility of XBJ with other intravenous drugs through Y-site. The quality and safety of co-administering XBJ with intravenous drugs remain unclear. This study investigates the physical compatibility of 53 commonly used intravenous drugs (31 Chinese medicine injections and 22 chemicals) with XBJ during Y-site administration. Methods used include visual inspection, Tyndall beam, particle limits, turbidity and pH changes, chromacity value changes, and spectroscopic absorption at 550 nm and 420 nm over 4 hours. Determining physical compatibility between XBJ and other intravenous drugs via Y-site could enhance safety management, reduce nursing time, and improve clinical utility. Additionally, we evaluated the sensitivity of these methods for scientific and feasible compatibility testing of Chinese herbal injections with other intravenous drugs to enhance infusion quality control.

## 2. Materials and methods

### 2.1 Sample preparation

Under aseptic and laminar air flow conditions, the selected 53 intravenous drugs were diluted in NS or D5W as recommended by the manufacturer, a total of 50 ml XBJ was slowly diluted in either NS or D5W to a concentration of 0.33 ml/ml. Selected drug solutions were slowly added into the diluted solution of XBJ, respectively. Each of sample solutions was passed through a 0.5 μm filter, XBJ solution and selected drug solutions were gradually combined during a single test, followed by mixing the resulting solutions with a plugged glass tube or an empty venous infusion bag at a 1:1 ratio. The final concentration of the mixed solutions was half that of a single drug.

All solutions were gently inverted three times at room temperature (approximately 22°C) at 0, 1, 2, and 4 h to ensure complete mixing. The solutions were then allowed to stand briefly or subjected to ultrasonic defoaming for 1 min. Physical compatibility including visual inspection, Tyndall beam, turbidity, particle counts, pH, chromacity value, A_550 nm_ and A_420 nm_ were observed and assessed intervals 0, 1, 2, and 4 h. Drug particulars, manufacturer, specification, lot number, diluent and concentrations were recorded for each drug (Table 1).

**Table 1:** Details of tested drugs in the study.

	Drug	Manufacturer	Specification	Lot	Diluent	Drug Concentration(/ml)
1	Aciclovir Sodium	Furen medicines group	0.25 g	2210237	NS	5mg
2	Ambroxol Hydrochloride	Huazhong Pharmaceutical	15mg/2ml	F221222A	NS	0.300 mg
3	Calcium Chloride	Sichuan Meida Kangjiale Pharmaceutical	0.5g/10ml	22062016	NS	5.000 mg
4	Calcium Gluconate	Sichuan Meida Kangjiale Pharmaceutical	1g/10ml	22121526	D5W	10.000 mg
5	Cefazolin Sodium	Zhejiang CR Sanjiu Zhongyi Pharmaceutical	0.5g	JX2204081	NS	10.000 mg
6	Cefoperazone Sodium and Sulbactam Sodium (1∶1)	Livzon Pharmaceutical Group	2g	DCK721201	NS	20.000 mg
7	Ceftriaxone Sodium	Hunan Kelun Pharmaceutical	1g	E2211014	NS	20.000 mg
8	Cefuroxime Sodium	Guangzhou Baiyunshan Tianxin Pharmaceutical	1.5g	2302384	NS	15.000 mg
9	Ciwujia injection	Duoduo Pharmaceutical	20ml	22061012	NS	1.000 mg
10	Composite Potassium Hydrogen Phosphate	Tianjin Jinyao Pharmaceutical	2ml	2209131	NS	0.005 ml
11	Danhong injection	Shandong Danhong Pharmaceutical	20ml	22082014	NS	0.140 ml
12	Danshen Chuanxiongqin injection	Jilin Sichang Pharmaceutical	5ml	20220709	NS	0.057 ml
13	Dazhu Hongjingtian injection	Tonghua Yusheng Pharmaceutical	5ml	1001220102	D5W	0.038 ml
14	Dengzhanxixin injection	Yunnan Biovalley Pharmaceutical	10ml	20220545	NS	0.074 ml
15	Dexamethasone Sodium Phosphate	Cisen Pharmaceutical	5mg/1ml	2205260611	NS	0.100 mg
16	Doxofylline	Zhejiang Beisheng Pharma Hansheng Pharmaceutical	0.3g/20ml	2101141	NS	3.000 mg
17	Extract of Ginkgo Biloba Leaves	Youcare Pharmaceutical Group	17.5mg/5ml	19821028	NS	0.350 mg
18	Ganciclovir sodium	Wuhan Pusheng Pharmaceutical	0.25g	230206–1	NS	5.000 mg
19	Ginkgo leaf Extract and Dipyridamole	Shanxi Pude Pharmaceutical	10ml	8220205	NS	0.048 ml
20	Guanxinning injection	Shineway Pharmaceutical	10ml	220320B3	NS	0.107 ml
21	Honghua injection	Shineway Pharmaceutical	10ml	220126D2	NS	0.074 ml
22	Huangqi injection	Shineway Pharmaceutical	10ml	210609A3	NS	0.074 ml
23	HuangqiDuotang for Injection	Tianjin Cinorch Pharmaceutical	250mg	220409	NS	1.000 mg
24	Hydrocortisone SodiumSuccinate	Yantai DongchengbeifangPharmaceutica	50mg	202208141	NS	1.000 mg
25	Ilaprazole Sodium	Livzon Pharmaceutical Group	10mg	210709	NS	0.100 mg
26	Kang’ai injection	Changbaishan Pharmaceutical	20ml	2220508	NS	0.140 ml
27	Kuhuang injection	Changshu Lei Yunshang Pharmaceutical	10ml	2206211	NS	0.108 ml
28	Lansoprazole Sodium	Aosaikang Pharmaceutical	30mg	J2201031	NS	0.300 mg
29	Magnesium Isoglycyrrhizinate	Chiatai Tianqing Pharmaceutical	50mg/10ml	221030204	NS	1.500 mg
30	Magnesium Sulfate	Shanghai Jindi Jiuzhou Pharmaceutical	2.5g/10ml	221041001C	D5W	25.000 mg
31	Mailuoning injection	Jinling Pharmaceutical	10ml	20220502	NS	0.074 ml
32	Methylprednisolone Sodium Succibate	Huapont Pharmaceutical	0.5g	230021212	NS	1.000 mg
33	OmeprazoleSodium	Huabei Pharmaceutical	40mg	2AFND20816	NS	0.400 mg
34	Pantoprazole Sodium	Yangtze River Pharmaceutical	40mg	22110141	NS	0.400 mg
35	Potassium Chloride	China Otsuka Pharmaceutical	1.5g/10ml	2G92K2	NS	3.000 mg
36	Qingkailing injection	Shineway Pharmaceutical	10ml	220126D2	NS	0.074 ml
37	Rabeprazole Sodium	Aosaikang Pharmaceutical	20mg	J2208081	NS	0.200 mg
38	Reduced Glutathione	YaoPharma	0.9g	22232450	NS	18.000 mg
39	Reduning injection	Jiangsu Kanion Pharmaceutical	10ml	220506	NS	0.074 ml
40	Shenfu injection	CR Sanjiu Ya’an Pharmaceutical	10ml	211101AK04	D5W	0.074 ml
41	Shenmai injection	Chiatai Qingchunbao Pharmaceutical	50ml	2202227	-	-
42	Shenqi Fuzheng injection	Livzon Group Limin Pharmaceutical	250ml	220537	-	-
43	Shuanghuanglian injection	Henan Fusen Pharmaceutical	20ml	2203211	NS	0.029 ml
44	Shuxuetong injection	Mudanjiang Youbo Pharmaceutical	2ml	22011202	NS	0.024 ml
45	Sulfotanshinone sodium	Shanghai Shangyao Diyishenghua Pharmaceutical	10mg/2ml	2303107	NS	0.160 mg
46	Tanreqing injection	Shanghai Kaibao Pharmaceutical	10ml	2206301	NS	0.074 ml
47	Xiangdan injection	Chiatai Qingchunbao Pharmaceutical	10ml	2203043	NS	0.074 ml
48	Xingnaojing injection	Wuxi Jiyushanhe Pharmaceutica	10ml	210807	NS	0.057 ml
49	Xinmailong injection	Yunnan Tengyao Pharmaceutica	2ml	2205101	NS	1.600 mg
50	Yinxingneizhi injection	Chengdu Baiyu Pharmaceutical	2ml	022110073	NS	0.200 mg
51	Xiyanping injection	Jiangxi Qinfeng Pharmaceutical	50mg/2ml	220714	NS	1.000 mg
52	Xuesaitong injection	Lonch GroupWanrong Pharmaceutical	20ml	22050211	D5W	1.600 mg
53	Xueshuantong for injection	Guangxi Wuzhou Pharmaceutical	150mg	22010321	NS	1.800 mg
54	XBJ	Tianjin Hongri Pharmaceutical	10ml	2302061	D5W	0.33ml
55	XBJ	Tianjin Hongri Pharmaceutical	10ml	2302061	NS	0.33ml
56	NS	Fengyuan Pharmaceutical	100ml	3122090602	-	-
57	D5W	Fengyuan Pharmaceutical	100ml	3122080801	-	-

### 2.2 Experimental controls

2 negative control solutions containing 0.33 ml/ml XBJ in D5W and 0.33 ml/ml XBJ in NS, 4 positive control solutions containing 2.5 mg/ml calcium chloride with 0.0025 ml/ml composite potassium in NS, 10 μm latex particles reference material and 25 μm particle count reference material (Haianhongmeng reference material technology Co., Ltd, Beijing, Lot: 20221003 and L693, respectively) were used as control solutions to ensure the test results in experiments.

### 2.3 Visual inspection

According to Part 4 of the Chinese Pharmacopoeia (Ch.P) 2020 edition, using the "Solution Color Test Method" and "Clarification Test Method" [10], at each test point, all solutions were examined using a clarity tester on both black and white backgrounds with the naked eye. Visual incompatibility was defined as color changes, gas evolution, or visible particulate formation within 4 hours.

### 2.4 Tyndall beam assessment

Tyndall beam was assessed using a red laser pointer (650 nm, 50 mW) on both black and white backgrounds. Any sample prevented the light from passing through the solutions and appearing Tyndall beam was considered incompatible [11, 12].

### 2.5 Turbidity measurement

Turbidity was measured at each time point through laboratory-grade turbidimeter (INESA Physico optical instrument Co., Ltd, Shanghai) according to the instructions. Three repeated measurements were taken for each sample, using the average as the final result. Physical incompatibility was defined as turbidity changes ≥0.5 NTU compared to 0 h [13, 14].

### 2.6 Particles measurement

Particle counts were counted using the GWF-8JA Particle Counter (Tianjin Tianhe Analysis Instrument Co., Ltd, Tianjin). Part 4 of the Ch.P recommended that injectable solutions be analyzed using a light obscuration particle count test. According to the Ch.P, for injectable solutions labeled as ≥100 ml, the number of ≥10 μm particles shall not exceed 25 particles/ml, the number of ≥25 μm particles shall not exceed 3 particles/ml [10]. Three repeated measurements were taken for each sample, using the average as the final result.

### 2.7 pH measurement

pH was measured to assess whether acid-base reactions may involve any observed incompatibilities. The same time points mentioned above (intervals 0, 1, 2, and 4 h), pH of solution were determined using a pH meter (Qiwei instrument Co., Ltd, Hangzhou). Any solutions with pH variations <10% compared to the baseline (immediately after mixing) were considered incompatible [15].

### 2.8 Chromacity value measurement

According to the Ch.P, "Solution Color Test Method" described using a colorimeter (Qiwei instrument Co., Ltd, Hangzhou) to determine chromacity value of the solutions [10]. It was stipulated to use yellow tonal standard solutions (Haianhongmeng reference material technology Co., Ltd, Beijing, Lot: M751) for comparison which contain 0.5, 1, 2, 3, 4, 5, 6, 7, 8, 9, 10 color codes. The chromacity value of 0.5 to 10 were about 25, 50, 100, 150, 200, 250, 300, 400, 500, 600, 700, respectively. If the chromacity value changes >200, and the color of the solution changes visually, solution color was defined as physical incompatibility.

### 2.9 Spectroscopic measurement

The Spectroscopic measurement was performed using 8453 Hewlett Packard diode array ultraviolet visible spectrophotometer to detect any indications of color change (A_420 nm_) or haze (A_550 nm_). All solutions were considered incompatible if the absorption changes by A_420 nm_ >0.0400 or A_550 nm_ >0.0100 [16].

### 2.10 Statistical analysis

Descriptive statistics and original data were presented. Results of particle count were reported as mean ± standard deviations (mean ± SD). No further statistical analysis was performed.

### 2.11 Definition of compatibility

**Fig 1:**
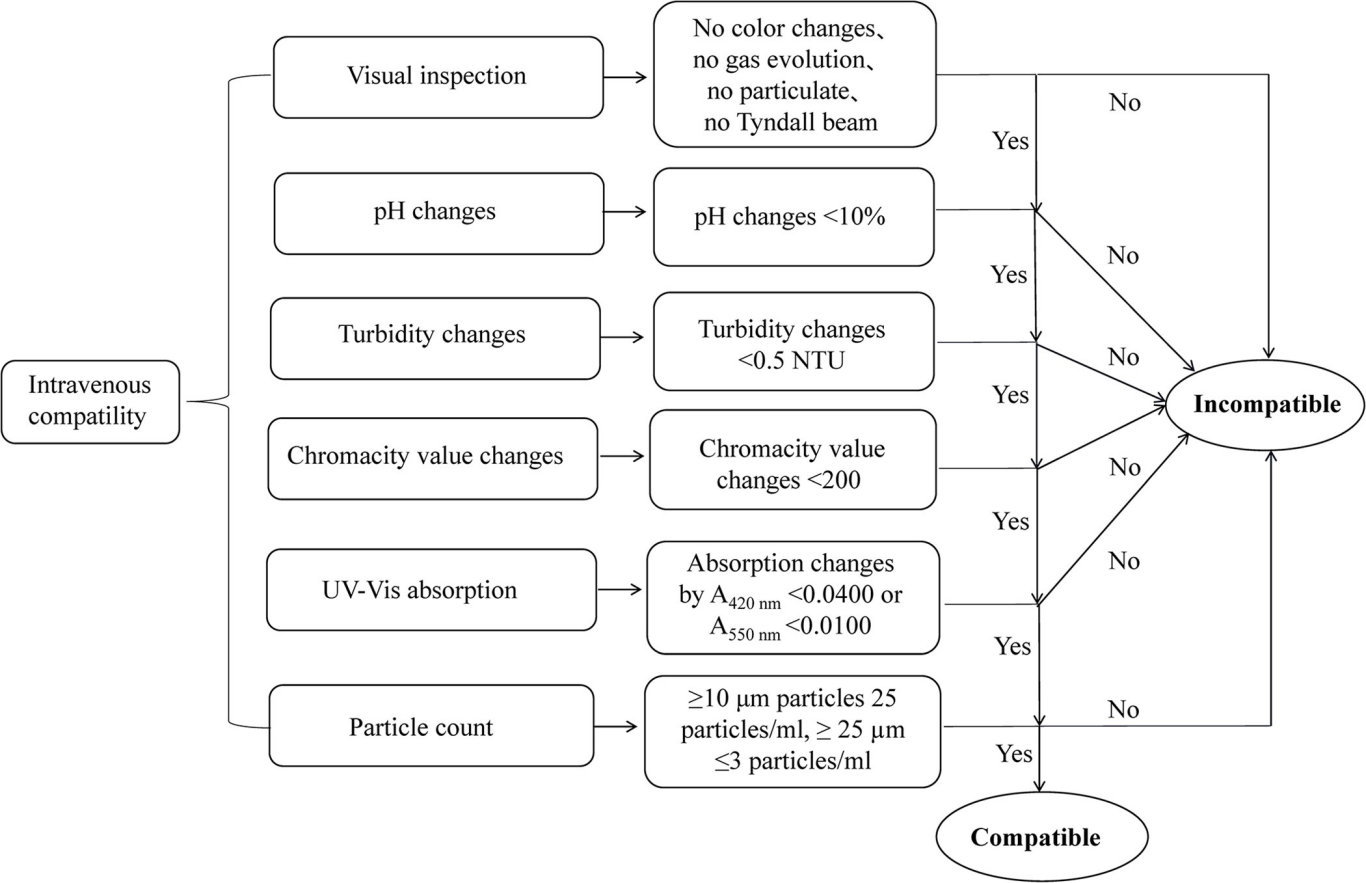
Diagram of physical compatibility justification.

Justification of compatibility refers to the diagram in Fig 1. Physical compatibility was defined as all solutions with no color changes, no gas evolution, particulate formation and no Tyndall beam within 4 hours, turbidity changes <0.5 NTU compared to 0 h, particle limits allowed over the Ch.P, pH changes <10% compared to 0, chromacity value changes <200 compared to 0 h, or absorption changes by A_420 nm_ >0.0400 or A_550 nm_ >0.0100 compared to 0 h.

## 3 Results

### 3.1 Visual inspection and Tyndall beam findings

**Fig 2:**
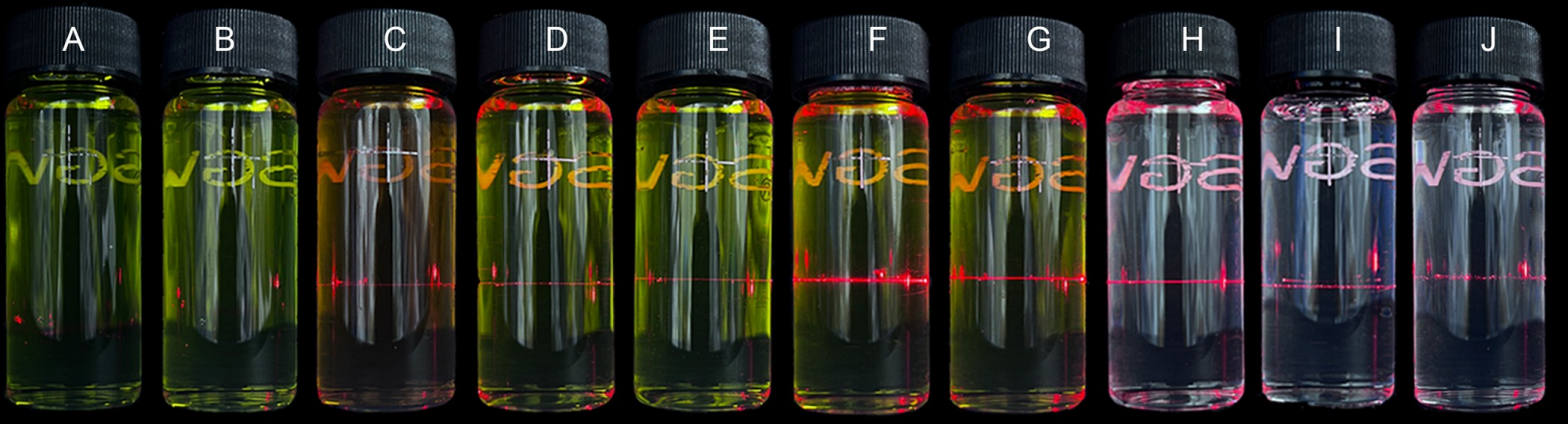
Tyndall beam of solutions. **Legend:** (A) Negative control, XBJ in D5W at 4 h. (B) Negative control, XBJ in NS at 4 h. (C) Combination of XBJ + Acyclovir Sodium in NS at 0 h. (D) Combination of XBJ + Ceftriaxone Sodium in NS at 0 h. (E) Combination of XBJ + Cefuroxime Sodium in NS at 0 h. (F) Combination of XBJ + Iprazole Sodium in NS at 0 h. (G) Combination of XBJ + Rabeprazole Sodium in NS at 0 h. (H) Positive control, Calcium Chloride with Composite Potassium Hydrogen Phosphate in NS at 0 h. (I) Positive control, 10 μm latex particles reference materials. (J) Positive control, 25 μm particle count reference materials.

No visual changes (color changes, gas evolution, haze, or visible particulate formation) were detected over the 4 hours period for the solutions (Table 2). However, the combination of XBJ + Acyclovir sodium, XBJ + Ceftriaxone sodium, XBJ + Cefuroxime sodium, XBJ + Iprazole sodium and XBJ + Rabeprazole sodium solutions in NS displayed Tyndall Beam, respectively (Fig 2), indicating that XBJ was incompatible with above 5 drugs.

**Table 2:** Findings of visual inspection, Tyndall beam, turbidity changes in solutions.

Drug	Color/Clarity (White background)				Tyndall beam (Black background)				Turbidity and the changes (NTU)				Compatibility
	0 h	1 h	2 h	4 h	0 h	1 h	2 h	4 h	0 h	1 h	2 h	4 h	
Aciclovir Sodium	Brown/Clear	Brown/Clear	Brown/Clear	Brown/Clear	P	P	P	P	0.202	0.047	-0.002	0.042	Incomp
Ambroxol Hydrochloride	Yellow/Clear	Yellow/Clear	Yellow/Clear	Yellow/Clear	N	N	N	N	0.061	0.031	0.025	0.021	Comp
Calcium Chloride	Yellow/Clear	Yellow/Clear	Yellow/Clear	Yellow/Clear	N	N	N	N	0.106	-0.049	0.026	-0.046	Comp
Calcium Gluconate	Yellow/Clear	Yellow/Clear	Yellow/Clear	Yellow/Clear	N	N	N	N	0.064	0.017	-0.014	0.013	Comp
Cefazolin Sodium	Yellow/Clear	Yellow/Clear	Yellow/Clear	Yellow/Clear	N	N	N	N	0.156	0.014	-0.022	-0.024	Comp
Cefoperazone Sodium and Sulbactam Sodium (1∶1)	Yellow/Clear	Yellow/Clear	Yellow/Clear	Yellow/Clear	N	N	N	N	0.096	-0.002	0.022	0.073	Comp
Ceftriaxone Sodium	Yellow/Clear	Yellow/Clear	Yellow/Clear	Yellow/Clear	P	P	P	P	0.135	0.145	-0.015	-0.007	Incomp
Cefuroxime Sodium	Yellow/Clear	Yellow/Clear	Yellow/Clear	Yellow/Clear	N	N	P	P	0.134	0.025	0.088	-0.007	Incomp
Ciwujia injection	Yellow/Clear	Yellow/Clear	Yellow/Clear	Yellow/Clear	N	N	N	N	0.106	-0.012	-0.014	-0.032	Comp
Composite Potassium Hydrogen Phosphate	Yellow/Clear	Yellow/Clear	Yellow/Clear	Yellow/Clear	N	N	N	N	0.056	0.035	0.022	0.019	Comp
Danhong injection	Yellow/Clear	Yellow/Clear	Yellow/Clear	Yellow/Clear	N	N	N	N	0.130	-0.042	-0.038	-0.046	Comp
Danshen Chuanxiongqin injection	Yellow/Clear	Yellow/Clear	Yellow/Clear	Yellow/Clear	N	N	N	N	0.111	0.003	0.016	-0.009	Comp
Dazhu Hongjingtian injection	Yellow/Clear	Yellow/Clear	Yellow/Clear	Yellow/Clear	N	N	N	N	0.066	-0.018	0.050	0.045	Comp
Dengzhanxixin injection	Yellow/Clear	Yellow/Clear	Yellow/Clear	Yellow/Clear	N	N	N	N	0.083	0.007	0.033	0.042	Comp
Dexamethasone Sodium Phosphate	Yellow/Clear	Yellow/Clear	Yellow/Clear	Yellow/Clear	N	N	N	N	0.109	0.029	0.056	0.000	Comp
Doxofylline	Yellow/Clear	Yellow/Clear	Yellow/Clear	Yellow/Clear	N	N	N	N	0.040	0.058	0.002	0.021	Comp
Extract of Ginkgo Biloba Leaves	Yellow/Clear	Yellow/Clear	Yellow/Clear	Yellow/Clear	N	N	N	N	0.122	-0.026	-0.006	-0.058	Comp
Ganciclovir sodium	Yellow/Clear	Yellow/Clear	Yellow/Clear	Yellow/Clear	N	N	N	N	0.131	-0.027	0.013	-0.009	Comp
Ginkgo leaf Extract and Dipyridamole	Yellow/Clear	Yellow/Clear	Yellow/Clear	Yellow/Clear	N	N	N	N	0.062	0.010	0.003	0.025	Comp
Guanxinning injection	Yellow/Clear	Yellow/Clear	Yellow/Clear	Yellow/Clear	N	N	N	N	0.145	-0.026	-0.048	-0.025	Comp
Honghua injection	Yellow/Clear	Yellow/Clear	Yellow/Clear	Yellow/Clear	N	N	N	N	0.066	0.030	0.067	-0.015	Comp
Huangqi injection	Yellow/Clear	Yellow/Clear	Yellow/Clear	Yellow/Clear	N	N	N	N	0.072	0.013	-0.007	0.008	Comp
HuangqiDuotang for Injection	Yellow/Clear	Yellow/Clear	Yellow/Clear	Yellow/Clear	N	N	N	N	0.219	0.041	0.088	0.065	Comp
Hydrocortisone SodiumSuccinate	Yellow/Clear	Yellow/Clear	Yellow/Clear	Yellow/Clear	N	N	N	N	0.105	-0.040	-0.049	-0.047	Comp
Ilaprazole Sodium	Yellow/Clear	Yellow/Clear	Yellow/Clear	Yellow/Clear	P	P	P	P	0.295	1.362	1.755	2.422	Incomp
Kang’ai injection	Yellow/Clear	Yellow/Clear	Yellow/Clear	Yellow/Clear	N	N	N	N	0.122	0.026	-0.059	-0.004	Comp
Kuhuang injection	Yellow/Clear	Yellow/Clear	Yellow/Clear	Yellow/Clear	N	N	N	N	0.159	-0.003	-0.023	-0.024	Comp
Lansoprazole Sodium	Yellow/Clear	Yellow/Clear	Yellow/Clear	Yellow/Clear	N	N	N	N	0.070	0.025	0.009	0.029	Comp
Magnesium Isoglycyrrhizinate	Yellow/Clear	Yellow/Clear	Yellow/Clear	Yellow/Clear	N	N	N	N	0.095	-0.041	-0.006	0.020	Comp
Magnesium Sulfate	Yellow/Clear	Yellow/Clear	Yellow/Clear	Yellow/Clear	N	N	N	N	0.075	0.046	-0.008	0.036	Comp
Mailuoning injection	Yellow/Clear	Yellow/Clear	Yellow/Clear	Yellow/Clear	N	N	N	N	0.105	0.008	0.005	0.001	Comp
Methylprednisolone Sodium Succibate	Yellow/Clear	Yellow/Clear	Yellow/Clear	Yellow/Clear	N	N	N	N	0.093	-0.016	-0.032	-0.023	Comp
OmeprazoleSodium	Yellow/Clear	Yellow/Clear	Yellow/Clear	Yellow/Clear	N	N	N	N	0.063	-0.002	-0.007	0.000	Comp
Pantoprazole Sodium	Yellow/Clear	Yellow/Clear	Yellow/Clear	Yellow/Clear	N	N	N	N	0.058	0.012	0.021	0.013	Comp
Potassium Chloride	Yellow/Clear	Yellow/Clear	Yellow/Clear	Yellow/Clear	N	N	N	N	0.037	0.049	0.036	0.073	Comp
Qingkailing injection	Yellow/Clear	Yellow/Clear	Yellow/Clear	Yellow/Clear	N	N	N	N	0.088	-0.008	-0.039	-0.005	Comp
Rabeprazole Sodium	Yellow/Clear	Yellow/Clear	Yellow/Clear	Yellow/Clear	P	P	P	P	0.222	1.533	4.270	6.773	Incomp
Reduced Glutathione	Yellow/Clear	Yellow/Clear	Yellow/Clear	Yellow/Clear	N	N	N	N	0.197	-0.003	-0.004	0.002	Comp
Reduning injection	Yellow/Clear	Yellow/Clear	Yellow/Clear	Yellow/Clear	N	N	N	N	0.062	0.009	-0.002	0.014	Comp
Shenfu injection	Yellow/Clear	Yellow/Clear	Yellow/Clear	Yellow/Clear	N	N	N	N	0.197	0.112	0.007	0.071	Comp
Shenmai injection	Yellow/Clear	Yellow/Clear	Yellow/Clear	Yellow/Clear	N	N	N	N	0.314	0.037	-0.040	-0.075	Comp
Shenqi Fuzheng injection	Yellow/Clear	Yellow/Clear	Yellow/Clear	Yellow/Clear	N	N	N	N	0.067	0.011	-0.006	0.002	Comp
Shuanghuanglian injection	Yellow/Clear	Yellow/Clear	Yellow/Clear	Yellow/Clear	N	N	N	N	0.093	-0.005	-0.014	-0.001	Comp
Shuxuetong injection	Yellow/Clear	Yellow/Clear	Yellow/Clear	Yellow/Clear	N	N	N	N	0.074	-0.021	0.040	0.130	Comp
Sulfotanshinone sodium	Red/Clear	Red/Clear	Red/Clear	Red/Clear	N	N	N	N	0.122	-0.013	-0.043	-0.022	Comp
Tanreqing injection	Yellow/Clear	Yellow/Clear	Yellow/Clear	Yellow/Clear	N	N	N	N	0.055	0.022	-0.015	0.084	Comp
Xiangdan injection	Yellow/Clear	Yellow/Clear	Yellow/Clear	Yellow/Clear	N	N	N	N	0.113	0.023	0.012	0.011	Comp
Xingnaojing injection	Yellow/Clear	Yellow/Clear	Yellow/Clear	Yellow/Clear	N	N	N	N	0.089	-0.010	-0.011	-0.012	Comp
Xinmailong injection	Yellow/Clear	Yellow/Clear	Yellow/Clear	Yellow/Clear	N	N	N	N	0.085	-0.017	-0.003	0.055	Comp
Yinxingneizhi injection	Yellow/Clear	Yellow/Clear	Yellow/Clear	Yellow/Clear	N	N	N	N	0.080	0.019	0.018	0.032	Comp
Xiyanping injection	Yellow/Clear	Yellow/Clear	Yellow/Clear	Yellow/Clear	N	N	N	N	0.100	0.013	-0.045	-0.036	Comp
Xuesaitong injection	Yellow/Clear	Yellow/Clear	Yellow/Clear	Yellow/Clear	N	N	N	N	0.084	0.014	-0.022	-0.036	Comp
Xueshuantong for injection	Yellow/Clear	Yellow/Clear	Yellow/Clear	Yellow/Clear	N	N	N	N	0.140	-0.064	-0.029	-0.027	Comp
XBJ^A^	Yellow/Clear	Yellow/Clear	Yellow/Clear	Yellow/Clear	N	N	N	N	0.102	0.008	-0.0013	-0.003	Comp
XBJ^B^	Yellow/Clear	Yellow/Clear	Yellow/Clear	Yellow/Clear	N	N	N	N	0.112	0.024	0.051	0.028	Comp
Calcium Chloride with Composite Potassium Hydrogen Phosphate^C^	White/Turbid	White Precipitate	White Precipitate	White Precipitate	P	P	P	P	3.405	67.825	-	-	Incomp
10 μm latex particles reference material^D^	Colourless/Clear				P				-	-	-	-	
25 μm particle count reference material^E^	Colourless/Clear				P				-	-	-	-	

**Note:** (A) Negative control, XBJ (Xuebijing injection) in D5W (dextrose 5% water). (B) Negative control, XBJ in NS (Normal saline). (C) Positive control, Calcium Chloride with Composite Potassium in NS. (D) Positive control, 10 μm latex particles reference material. (E) Positive control, 25 μm particle count reference material, P; Tyndall Positive, N; Tyndall Negative, Comp; compatible, Incomp; incompatible

### 3.2 Turbidity changes findings

The results from turbidity measurement were summarized in Table 2. Of the 53 selected drugs, combinations of XBJ + 51 drugs result in turbidity changes <0.5 NTU compared to 0 h, respectively. At the time of 1, 2, 4 h, combinations of XBJ + Iprazole sodium and XBJ + Rabeprazole sodium solutions displayed turbidity changes >0.5 NTU compared to 0 h. It indicated that XBJ was incompatible with Iprazole sodium and Rabeprazole sodium.

### 3.3 Particle count findings

Table 3 showed the results of the particle count findings. After mixing, a massive growth in number of particles in group of XBJ with Danshen Chuanxiongqin injection, Dengzhanxixin injection, Guanxinning injection, Kang’ai injection, Kuhuang injection, Qingkailing injection, Shenfu injection, Xingnaojing injection, Xueshuantong for injection, Aciclovir sodium, Cefoperazone sodium and Sulbactam sodium (1:1), Ceftriaxone sodium, Cefuroxime sodium, Composite Potassium Hydrogen Phosphate, Ganciclovir sodium, Ilaprazole sodium, Rabeprazole sodium, respectively. Then showed a increase within the 0 to 4 h in our study. The particles in above solutions exceed the recommended the limitation of the Ch.P specification. At the time of 4 h particles in group of XBJ + Tanreqing injection and XBJ + Cefazolin sodium also exceed the limitation of the Ch.P, indicating that XBJ was incompatible with above 19 drugs.

**Table 3:** Results of particle count and pH changes of solutions.

Drug	≥10 μm Particles/ml (SD)				≥25 μm Particles/ml (SD)				pH and the changes				Compatibility
	0 h	1 h	2 h	4 h	0 h	1 h	2 h	4 h	0 h	1 h	2 h	4 h	
Aciclovir Sodium	52.70(0.68)	89.01(2.00)	92.36(0.89)	57.44(4.22)	2.22(0.15)	4.29(0.25)	3.29(0.19)	3.51(0.14)	9.71	0.01	0.01	-0.04	Incomp
Ambroxol Hydrochloride	11.56(3.74)	12.22(10.58)	11.60(1.85)	15.09(14.11)	0.29(0.20)	0.71(0.94)	0.36(0.10)	0.36 (0.33)	5.68	0.17	0.08	0.04	Comp
Calcium Chloride	6.36(1.35)	20.24(1.20)	10.04(2.88)	17.82(4.17)	0.29(0.04)	1.11(0.10)	0.73(0.18)	0.49(0.44)	5.52	-0.08	0.25	0.08	Comp
Calcium Gluconate	5.33(5.84)	6.53(2.17)	17.49(8.04)	5.87(0.47)	0.36(0.08)	0.11(0.08)	1.67(1.72)	2.53(3.01)	5.22	0.04	0.02	0.05	Comp
Cefazolin Sodium	10.98(6.00)	18.71(3.88)	18.80(7.88)	28.98(7.47)	0.31(0.10)	0.58(0.10)	0.82(0.48)	0.80(0.35)	5.14	-0.13	-0.08	-0.08	Incomp
Cefoperazone Sodium and Sulbactam Sodium (1∶1)	53.22(2.67)	69.40(2.27)	43.22(1.27)	56.36(4.35)	1.02(0.04)	2.84(1.80)	1.27(0.12)	2.33(0.12)	4.96	-0.07	-0.09	-0.08	Incomp
Ceftriaxone Sodium	35.16(0.60)	32.07(0.61)	49.98(0.50)	110.93(1.20)	0.87(0.27)	0.89(0.17)	2.04(0.10)	3.64(0.15)	5.69	0.16	0.13	0.15	Incomp
Cefuroxime Sodium	32.96(0.52)	56.36(0.44)	114.40(1.39)	137.58(0.43)	0.60(0.12)	1.69(0.08)	4.36(0.34)	7.53(0.12)	5.75	-0.51	-0.52	-0.47	Incomp
Ciwujia injection	18.38(0.38)	20.49(2.87)	12.98(2.30)	14.24(1.01)	1.89(0.10)	1.02(0.23)	0.73(0.18)	1.56(0.08)	5.16	-0.02	0.00	0.03	Comp
Composite Potassium Hydrogen Phosphate	25.73(15.00)	33.04(19.3)	26.40(19.54)	25.29(6.73)	0.31(0.15)	1.56(0.14)	1.62(2.12)	0.84(0.67)	7.04	0.07	0.08	0.07	Incomp
Danhong injection	13.56(2.01)	11.38(3.33)	7.64(3.83)	19.80(14.52)	0.40(0.13)	0.87(0.52)	0.36(0.23)	0.53(0.35)	5.77	-0.11	0.00	-0.05	Comp
Danshen Chuanxiongqin injection	48.27(20.09)	51.98(9.34)	25.29(4.39)	125.53(27.26)	1.64(0.32)	0.87(0.31)	0.51(0.28)	5.11(0.63)	4.15	-0.13	-0.10	0.03	Incomp
Dazhu Hongjingtian injection	3.89(0.91)	5.87(0.59)	21.11(0.20)	2.11(0.23)	0.91(0.14)	0.59(0.10)	0.20(0.14)	0.23(0.13)	5.26	0.03	0.01	0.06	Comp
Dengzhanxixin injection	40.07(3.87)	29.20(1.11)	37.44(1.05)	50.64(6.58)	0.56(0.04)	0.51(0.10)	0.76(0.04)	3.73(0.32)	5.38	0.05	0.03	0.03	Incomp
Dexamethasone Sodium Phosphate	11.98(1.82)	13.18(6.63)	7.62(2.33)	8.56(1.65)	0.33(0.18)	0.71(0.32)	0.33(0.12)	0.20(0.07)	6.27	-0.06	-0.05	-0.07	Comp
Doxofylline	10.80(6.58)	13.71(3.93)	15.02(5.94)	9.33(5.90)	0.56(0.44)	1.18(0.94)	0.11(0.19)	0.29(0.14)	5.92	0.23	-0.09	-0.12	Comp
Extract of Ginkgo Biloba Leaves	16.44(0.17)	12.78(0.42)	14.18(0.37)	6.02(1.42)	1.2(0.92)	0.36(0.14)	0.64(0.33)	0.49(0.10)	6.49	-0.08	-0.23	-0.22	Comp
Ganciclovir sodium	10.58(0.77)	67.11(1.44)	75.22(1.29)	35.27(2.02)	0.42(0.23)	3.56(0.08)	3.29(0.14)	3.62(2.21)	9.31	0.01	0.05	-0.33	Incomp
Ginkgo leaf Extract and Dipyridamole	12.20(0.00)	9.02(5.70)	11.04(5.96)	13.22(3.61)	0.69(0.56)	0.47(0.64)	0.80(0.76)	0.89(0.86)	5.35	0.02	0.02	-0.04	Comp
Guanxinning injection	30.67(1.67)	33.28(10.11)	44.02(4.29)	27.62(4.53)	0.80(0.35)	0.87(0.27)	1.27(0.47)	0.98(0.80)	5.59	0.08	0.05	0.11	Incomp
Honghua injection	9.93(3.82)	11.31(7.03)	7.31(0.04)	24.04(5.34)	0.27(0.12)	0.96(0.60)	0.58(0.21)	1.78(0.31)	5.66	0.24	0.06	0.07	Comp
Huangqi injection	15.36(3.77)	10.67(3.47)	7.49(2.72)	15.27(3.99)	0.71(0.39)	0.51(0.08)	0.31(0.04)	1.18(0.20)	6.71	-0.18	-0.18	-0.26	Comp
HuangqiDuotang for Injection	19.24(0.71)	17.80(0.58)	11.27(1.62)	6.00(0.69)	1.67(0.37)	0.93(0.46)	1.58(1.00)	0.22(0.08)	5.61	0.19	0.13	0.02	Comp
Hydrocortisone SodiumSuccinate	16.93(7.51)	13.38(8.69)	7.42(6.44)	9.91(7.19)	0.69(0.91)	0.71(0.42)	0.16(0.21)	0.67(0.69)	6.36	-0.10	-0.13	-0.11	Comp
Ilaprazole Sodium	56.33(14.57)	89.80(59.03)	84.76(12.98)	37.67(11.81)	3.09(1.89)	3.36(3.62)	3.49(3.76)	3.20(0.80)	6.37	-0.04	-0.11	-0.09	Incomp
Kang’ai injection	35.96(12.29)	34.36(19.11)	29.08(10.03)	85.89(6.93)	0.89(0.28)	0.69(1.14)	0.74(1.00)	3.67(1.75)	6.71	0.02	-0.08	-0.01	Incomp
Kuhuang injection	66.80(21.32)	78.98(0.77)	43.56(2.17)	38.91(8.61)	2.20(0.44)	0.78(0.25)	0.82(0.19)	0.62(0.15)	5.74	-0.10	-0.09	-0.03	Incomp
Lansoprazole Sodium	17.00(0.77)	13.91(0.28)	7.44(0.38)	9.82(0.19)	1.47(0.13)	0.80(0.18)	0.47(0.35)	0.49(0.04)	7.69	0.05	0.02	-0.09	Comp
Magnesium Isoglycyrrhizinate	15.60(7.68)	10.38(7.18)	10.51(5.92)	13.27(9.65)	0.38(0.23)	0.40(0.37)	0.42(0.41)	0.28(0.09)	6.21	0.05	-0.07	0.10	Comp
Magnesium Sulfate	12.04(6.05)	23.91(22.46)	8.33(4.87)	8.91(7.73)	1.09(1.35)	0.31(0.42)	0.18(0.10)	1.11(1.75)	5.62	0.01	-0.21	-0.23	Comp
Mailuoning injection	11.98(1.46)	17.73(13.58)	8.87(1.92)	7.42(0.27)	0.48(0.20)	0.49(0.34)	0.31(0.34)	0.58(0.37)	6.30	0.13	-0.07	0.06	Comp
Methylprednisolone Sodium Succibate	14.87(6.09)	23.45(14.55)	17.22(12.65)	9.02(6.45)	0.98(0.60)	0.74(0.63)	0.98(0.62)	0.60(0.47)	7.42	-0.18	-0.11	-0.08	Comp
OmeprazoleSodium	5.07(1.51)	7.84(1.81)	15.11(3.07)	10.58(6.20)	0.31(0.14)	0.38(0.10)	0.42(0.20)	0.18(0.17)	7.94	0.05	0.08	0.02	Comp
Pantoprazole Sodium	15.69(9.76)	8.38(2.04)	10.27(4.56)	7.67(3.18)	0.40(0.29)	0.18(0.19)	0.31(0.17)	0.27(0.29)	7.33	0.13	0.18	0.05	Comp
Potassium Chloride	7.44(1.04)	4.02(1.65)	17.40(3.47)	14.82(5.79)	0.33(0.18)	0.18(0.08)	1.27(0.47)	1.24(0.93)	5.35	0.36	0.22	0.49	Comp
Qingkailing injection	28.58(1.60)	29.33(0.48)	25.78(3.04)	30.07(0.98)	2.11(0.23)	2.00(0.18)	1.38(1.07)	2.09(0.04)	6.24	0.08	0.18	-0.01	Incomp
Rabeprazole Sodium	43.20(29.84)	116.22(110.53)	93.89(59.61)	71.02(52.03)	2.67(1.10)	5.51(5.13)	6.27(4.91)	3.04(2.32)	6.87	0.04	0.00	-0.13	Incomp
Reduced Glutathione	8.49(2.33)	6.67(0.88)	6.89(1.83)	17.71(7.86)	0.44(0.10)	0.18(0.17)	0.31(0.43)	0.96(1.16)	5.69	-0.03	-0.04	-0.02	Comp
Reduning injection	9.49(2.35)	16.27(1.81)	10.40(5.06)	16.98(0.99)	0.69(0.10)	0.73(0.18)	0.20(0.07)	1.07(0.29)	4.78	-0.03	-0.02	-0.03	Comp
Shenfu injection	33.31(2.49)	30.53(1.83)	88.6(6.4)	87.47(4.67)	1.62(0.19)	0.44(0.10)	1.53(0.13)	2.82(0.15)	5.79	0.08	0.08	0.19	Incomp
Shenmai injection	8.76(0.34)	8.98(4.52)	4.07(1.77)	18.96(3.74)	0.27(0.07)	0.56(0.14)	0.36(0.15)	0.53(0.29)	5.25	0.02	0.06	0.10	Comp
Shenqi Fuzheng injection	6.62(4.31)	11.16(1.46)	16.84(0.62)	6.84(1.08)	0.11(0.19)	0.53(0.12)	0.93(0.58)	0.53(0.46)	5.58	0.23	0.00	0.03	Comp
Shuanghuanglian injection	17.07(0.47)	19.53(0.57)	11.11(1.44)	15.47(0.84)	0.89(0.17)	1.24(0.08)	0.42(0.14)	0.76(0.17)	5.21	0.10	0.10	-0.01	Comp
Shuxuetong injection	6.91(2.57)	9.62(0.47)	6.76(2.08)	7.60(2.48)	0.58(0.10)	0.56(0.20)	0.36(0.10)	0.33(0.13)	5.14	-0.01	0.00	0.03	Comp
Sulfotanshinone sodium	8.80(2.27)	4.73(2.50)	9.67(2.66)	11.73(4.97)	0.44(0.21)	0.18(0.14)	0.27(0.18)	0.49(0.44)	5.50	0.03	0.01	-0.01	Comp
Tanreqing injection	14.87(4.47)	12.09(2.14)	12.8(1.44)	41.16(7.02)	0.38(0.20)	0.42(0.17)	0.49(0.17)	1.84(0.57)	6.40	0.20	0.02	0.02	Incomp
Xiangdan injection	17.76(6.80)	14.38(3.22)	10.69(0.74)	14.44(5.35)	1.27(0.72)	0.67(0.57)	0.29(0.04)	0.69(0.27)	5.57	0.14	0.14	0.09	Comp
Xingnaojing injection	9.87(3.03)	6.27(3.23)	51.73(23.98)	39.33(4.24)	0.42(0.14)	0.87(0.72)	2.60(0.75)	2.96(0.21)	5.73	-0.03	-0.23	-0.20	Incomp
Xinmailong injection	13.31(1.85)	6.47(4.72)	7.60(3.07)	8.18(1.10)	0.73(0.12)	0.40(0.20)	0.20(0.07)	0.29(0.17)	5.64	-0.08	-0.10	0.06	Comp
Yinxingneizhi injection	19.24(0.92)	16.16(0.76)	20.07(0.29)	13.04(1.77)	1.24(0.04)	1.11(0.10)	0.93(0.00)	1.31(0.08)	5.51	-0.05	0.01	0.10	Comp
Xiyanping injection	7.56(2.27)	11.69(4.85)	12.36(11.71)	7.60(2.91)	0.38(0.25)	0.31(0.37)	0.18(0.25)	0.13(0.00)	5.73	-0.04	-0.05	-0.19	Comp
Xuesaitong injection	8.36(0.91)	18.58(7.71)	14.73(0.66)	14.96(5.63)	0.38(0.17)	1.62(0.25)	1.11(0.10)	0.84(0.44)	5.41	0.17	0.26	0.12	Comp
Xueshuantong for injection	53.13(6.13)	27.87(2.08)	27.02(0.28)	46.73(4.20)	1.87(0.07)	0.71(0.10)	1.42(0.08)	2.40(0.20)	5.06	0.03	-0.14	0.01	Incomp
XBJ^A^	20.11(2.93)	10.87(0.31)	15.51(0.30)	8.60(0.24)	0.64(0.37)	0.93(0.35)	1.51(0.51)	0.73(0.13)	5.53	0.02	0.01	0.03	Comp
XBJ^B^	13.98(2.54)	18.67(1.65)	14.29(0.57)	20.33(0.42)	0.44(0.20)	1.56(0.34)	1.53(0.37)	0.96(0.25)	6.26	0.00	0.00	0.00	Comp
Calcium Chloride with Composite Potassium Hydrogen Phosphate^C^	192.07(56.11)	41257.73(499.97)	-	-	17.47(14.40)	146.60(12.69)	-	-	-				Incomp
10 μm latex particles reference material^D^	1236(4.20)				-				-				Incomp
25 μm particle count reference material^E^	-				1973(3.87)				-				Incomp

**Note:** (A) Negative control, XBJ (Xuebijing injection) in D5W (dextrose 5% water). (B) Negative control, XBJ in NS (Normal saline). (C) Positive control, Calcium Chloride with Composite Potassium in NS. (D) Positive control, 10 μm latex particles reference material. (E) Positive control, 25 μm particle count reference material, Comp; compatible, Incomp; incompatible

### 3.4 pH changes findings

Table 3 exhibited pH range of combinations of XBJ with other intravenous drugs (4.0 to 10.0). pH changes that did not vary >10% from baseline (immediately after mixing) in any admixture.

### 3.5 Chromacity value changes findings

Table 4 exhibited XBJ had no change in color visually and chromacity value ≥700 within 4 h with the selected 53 intravenous drugs mixing solutions. It inferred that the chromacity value change of 1, 2, 4 h were <200 compared to 0 h, two drugs were physically compatible.

**Table 4:** Results of chromacity value, photometrical changes of solutions.

Drug	Chromacity value				A_550 nm_				A_420 nm_				Compatibility
`	0 h	1 h	2 h	4 h	0 h	1 h	2 h	4 h	0 h	1 h	2 h	4 h	
Aciclovir Sodium	≥700	≥700	≥700	≥700	0.1613	-0.0030	-0.0034	-0.0109	3.4149	0.3867	-0.1297	-0.2893	Incomp
Ambroxol Hydrochloride	≥700	≥700	≥700	≥700	0.0080	0.0023	0.0025	0.0058	3.4153	0.0188	-0.0082	-0.2460	Comp
Calcium Chloride	≥700	≥700	≥700	≥700	0.0270	-0.0016	0.0037	-0.0012	3.0196	0.1606	0.2599	0.0280	Comp
Calcium Gluconate	≥700	≥700	≥700	≥700	0.0377	0.0030	-0.0026	0.0013	3.1794	0.1750	-0.0898	-0.0565	Comp
Cefazolin Sodium	≥700	≥700	≥700	≥700	0.0386	-0.0078	-0.0084	-0.0016	3.0707	0.4705	0.3936	-0.1086	Comp
Cefoperazone Sodium and Sulbactam Sodium (1∶1)	≥700	≥700	≥700	≥700	0.0300	0.0032	-0.0015	0.0040	3.0079	0.2756	0.4249	0.9797	Comp
Ceftriaxone Sodium	≥700	≥700	≥700	≥700	0.0518	-0.0144	-0.0139	-0.0391	3.2581	-0.0954	-0.3711	-0.1984	Incomp
Cefuroxime Sodium	≥700	≥700	≥700	≥700	0.0366	0.0117	-0.0132	-0.0353	3.1501	0.5438	0.0161	-0.1463	Incomp
Ciwujia injection	≥700	≥700	≥700	≥700	0.1388	-0.0067	-0.0004	0.0026	4.0643	-0.6629	-0.8177	-0.9129	Comp
Composite Potassium Hydrogen Phosphate	≥700	≥700	≥700	≥700	0.0404	0.0034	-0.0005	0.0089	3.2262	-0.0030	0.2350	0.0297	Comp
Danhong injection	≥700	≥700	≥700	≥700	0.0771	-0.0010	-0.0087	-0.0092	3.3121	0.1410	0.2379	-0.1409	Comp
Danshen Chuanxiongqin injection	≥700	≥700	≥700	≥700	0.0171	0.0040	0.0222	-0.0148	3.1010	0.0152	0.1192	0.2142	Incomp
Dazhu Hongjingtian injection	≥700	≥700	≥700	≥700	0.0295	0.0033	0.0015	0.0024	3.3764	-0.1408	-0.2946	-0.2731	Comp
Dengzhanxixin injection	≥700	≥700	≥700	≥700	0.0556	0.0005	-0.0003	-0.0036	3.2579	-0.1641	-0.2153	-0.0647	Comp
Dexamethasone Sodium Phosphate	≥700	≥700	≥700	≥700	0.0209	-0.0007	0.0002	-0.0015	3.0749	0.0419	0.2617	0.1608	Comp
Doxofylline	≥700	≥700	≥700	≥700	0.0113	0.0008	0.0063	0.0116	3.1805	0.2562	0.1806	-0.1239	Incomp
Extract of Ginkgo Biloba Leaves	≥700	≥700	≥700	≥700	0.0468	0.0039	-0.0090	-0.0132	3.1753	0.6972	0.6402	0.2115	Incomp
Ganciclovir sodium	≥700	≥700	≥700	≥700	0.1296	0.0137	0.0148	0.0198	3.6969	-0.4958	-0.8039	-0.9113	Incomp
Ginkgo leaf Extract and Dipyridamole	≥700	≥700	≥700	≥700	0.0292	-0.0090	-0.0016	0.0012	3.4612	-0.1975	-0.2302	-0.2526	Comp
Guanxinning injection	≥700	≥700	≥700	≥700	0.0531	0.0115	0.0200	0.0276	3.4168	-0.1032	-0.0110	-0.1160	Incomp
Honghua injection	≥700	≥700	≥700	≥700	0.0668	-0.0093	-0.0007	0.0029	3.5088	-0.0843	-0.2431	-0.2447	Comp
Huangqi injection	≥700	≥700	≥700	≥700	0.0564	0.0062	-0.0098	0.0013	3.4831	-0.3366	-0.2205	-0.3444	Comp
Huangqi Duotang for Injection	≥700	≥700	≥700	≥700	0.0265	0.0099	-0.0040	-0.0006	3.1719	0.1836	0.0303	0.1164	Comp
Hydrocortisone Sodium Succinate	≥700	≥700	≥700	≥700	0.0323	0.0004	0.0036	0.0065	3.2429	0.2774	0.2582	0.1278	Comp
Ilaprazole Sodium	≥700	≥700	≥700	≥700	0.0514	0.0245	0.0327	0.0407	3.1529	0.2611	0.6093	0.0532	Incomp
Kang’ai injection	≥700	≥700	≥700	≥700	0.0189	0.0129	0.0202	0.0125	3.2428	-0.1354	-0.1632	-0.1913	Incomp
Kuhuang injection	≥700	≥700	≥700	≥700	0.0517	0.0250	0.0244	0.0285	3.1223	-0.0929	-0.0834	-0.0620	Incomp
Lansoprazole	≥700	≥700	≥700	≥700	0.0361	0.0125	0.0229	0.0177	3.6358	-0.1696	-0.0175	0.0663	Incomp
Magnesium Isoglycyrrhizinate	≥700	≥700	≥700	≥700	0.0184	-0.0022	-0.0042	-0.0023	3.3400	0.0524	0.0403	-0.0604	Comp
Magnesium Sulfate	≥700	≥700	≥700	≥700	0.0308	0.0030	0.0082	0.0106	3.1031	0.1857	0.2559	0.0463	Incomp
Mailuoning injection	≥700	≥700	≥700	≥700	0.0505	0.0040	0.0018	-0.0052	3.4737	0.3838	0.3483	0.0360	Comp
Methylprednisolone Sodium Succibate	≥700	≥700	≥700	≥700	0.0455	-0.0002	0.0025	0.0042	3.4922	0.1789	-0.0243	-0.0214	Comp
Omeprazole Sodium	≥700	≥700	≥700	≥700	0.0573	-0.0228	-0.0169	0.0130	3.5021	-0.2590	-0.3366	-0.4169	Incomp
Pantoprazole Sodium	≥700	≥700	≥700	≥700	0.0517	-0.0227	-0.0260	-0.0115	3.4664	-0.0328	-0.2284	-0.3333	Incomp
Potassium Chloride	≥700	≥700	≥700	≥700	0.0156	0.0028	-0.0016	-0.0008	2.9599	0.1368	0.1969	0.0247	Comp
Qingkailing injection	≥700	≥700	≥700	≥700	0.0486	0.0024	0.0002	0.0115	3.3089	-0.0117	-0.0236	-0.1111	Incomp
Rabeprazole Sodium	≥700	≥700	≥700	≥700	1.1166	0.4382	1.0674	1.8095	3.1098	0.2287	0.2870	0.4238	Incomp
Reduced Glutathione	≥700	≥700	≥700	≥700	0.0299	0.0031	-0.0068	-0.0042	3.4335	0.0052	0.0303	-0.1220	Comp
Reduning injection	≥700	≥700	≥700	≥700	0.0596	0.0047	0.0057	0.0075	3.3104	0.5311	0.3123	0.1935	Comp
Shenfu injection	≥700	≥700	≥700	≥700	0.0407	-0.0089	-0.0058	-0.0009	3.2368	0.0442	0.0310	-0.0339	Comp
Shenmai injection	≥700	≥700	≥700	≥700	0.0446	0.0018	0.0016	-0.0014	3.2116	0.3510	0.5023	-0.0759	Comp
Shenqi Fuzheng injection	≥700	≥700	≥700	≥700	0.0312	-0.0025	-0.0046	-0.0002	3.2320	0.7015	0.3855	0.1261	Comp
Shuanghuanglian injection	≥700	≥700	≥700	≥700	0.0834	0.0022	-0.0072	0.0024	3.6586	-0.0024	0.2875	-0.0681	Comp
Shuxuetong injection	≥700	≥700	≥700	≥700	0.0475	-0.0047	-0.0032	-0.0037	3.2062	0.5964	0.2515	0.0008	Comp
Sulfotanshinone sodium	≥700	≥700	≥700	≥700	0.2261	0.0069	0.0043	0.0032	3.8027	-0.4262	-0.4684	-0.6765	Comp
Tanreqing injection	≥700	≥700	≥700	≥700	0.0767	0.0139	-0.0308	0.0119	3.3278	0.2529	0.1107	-0.0549	Incomp
Xiangdan injection	≥700	≥700	≥700	≥700	0.0537	-0.0055	-0.0219	-0.0184	3.2809	-0.0320	-0.0320	-0.0521	Incomp
Xingnaojing injection	≥700	≥700	≥700	≥700	0.0264	0.0076	0.0078	0.0049	3.2257	0.3063	0.2352	0.2463	Comp
Xinmailong injection	≥700	≥700	≥700	≥700	0.0312	-0.0092	-0.0033	0.0005	3.2165	0.2327	0.3796	-0.0130	Comp
Yinxingneizhi injection	≥700	≥700	≥700	≥700	0.0291	0.0039	0.0082	0.0136	3.1696	-0.0444	-0.0700	-0.0423	Incomp
Xiyanping injection	≥700	≥700	≥700	≥700	0.0258	0.0017	0.0056	0.0009	3.2403	0.6899	0.4666	0.0525	Comp
Xuesaitong injection	≥700	≥700	≥700	≥700	0.0323	0.0038	0.0066	0.0028	3.2665	0.2646	0.0040	-0.0851	Comp
Xueshuantong for injection	≥700	≥700	≥700	≥700	0.0080	0.0414	0.0512	0.0525	2.8011	0.0578	0.0425	0.2291	Incomp
XBJ^A^	≥700	≥700	≥700	≥700	0.0875	-0.0058	-0.0097	-0.0087	3.3454	0.3226	0.2888	0.3435	Comp
XBJ^B^	≥700	≥700	≥700	≥700	0.0581	-0.0023	-0.0043	0.0032	3.5740	-0.0799	-0.2953	-0.3025	Comp

**Note:** (A) Negative control, XBJ (Xuebijing injection) in D5W (dextrose 5% water). (B) Negative control, XBJ in NS (Normal saline), Comp; compatible, Incomp; incompatible

### 3.6 Photometrical changes findings

Absorption at 550 nm were detected. Binary combinations of XBJ with Danshen Chuanxiongqin injection, Guanxinning injection, Kang’ai injection, Kuhuang injection, Tanreqing injection, Xiangdan injection, Xueshuantong for injection, Ceftriaxone sodium, Cefuroxime sodium, Ganciclovir sodium, Ilaprazole sodium, Lansoprazole sodium, Omeprazole sodium, Pantoprazole sodium, Rabeprazole sodium solutions immediately displayed A_550 nm_ changes >0.0100 compared to 0 h and combinations of XBJ with Extract of Ginkgo Biloba Leaves, Qingkailing injection, Yinxingneizhi injection, Aciclovir sodium, Doxofylline, Magnesium Sulfate solutions displayed A_550 nm_ changes >0.0100 at 4 h compared to 0 h (Table 4), the results showed that XBJ was incompatible with above 21 drugs.

Binary combinations of XBJ with 53 intravenous drug solutions were primarily yellow or brown-yellow in appearance. The range of A_420 nm_ varied from 2.8000 to 4.1000, resulting in a significant error. The change in A_420 nm_ exceeded 0.0400 (Table 4).

## 4. Discussion

XBJ infusion was usually administered clinically within 4 hours. In this study, the physical compatibility for XBJ in binary combinations with selected 53 intravenous drugs were evaluated. Table 2 showed that no visual changes (color changes, gas evolution, haze, or visible particulate formation) were detected over the 4 hours period for the solutions. However, the combination of XBJ +Acyclovir solutions, XBJ + Ceftriaxone sodium, XBJ + Cefuroxime sodium, XBJ + Iprazole sodium and XBJ + Rabeprazole sodium solutions in NS produced Tyndall Beam respectively (Fig 2), indicating that XBJ was incompatible with above 5 drugs. Since the discovery of the Tyndall effect, it has been mainly used to assist the observation of the effect of colloidal solutions in experiments. As a control index of solution properties for intravenous drugs, Tyndall effect can directly reflect the solution quality and physical compatibility of intravenous drugs without damaging the package of drug solutions [11, 12, 17]. In our study, three inspections of color change, clarification and Tyndall effect were used to evaluate the properties of solutions, and it was compared with 10 μm latex particles material, 25 μm particle count reference material and mixed solution of Calcium Chloride with Composite Potassium, which could produce Tyndall effect. It was proved that Tyndall effect could be used as an inspection method of solution properties and physical compatibility of Chinese medicine injection. Three inspections can objectively reflect the quality of the solution of Chinese medicine injection. However, our studies found that oily Chinese medicine fat milk injections such as Brucea Javanica oil emulsion injection, Elemene injectable emulsion and Zedoray Turmeric oil injection can produce Tyndall beam, which is not suitable for this inspection method. The inspection of these kind of intravenous drug solutions need further study in our research.

The turbidity of the solutions can be measured by a turbidity meter. Different size and qualitative of particle matter in solutions can scatter the incident light. The turbidity of the solutions can be checked by measuring the intensity of the transmitted or scattered light. The transmitted-scattered light comparative measurement model is used for the turbidity determination of low and medium turbidity colorless solutions (turbidity below 100 NTU). The colored substance may reduce the turbidity of the solution, but yellow has the least effect on the results [18]. The diluted solution of XBJ was a yellow solution, 31 kinds of traditional Chinese medicine injection were mainly yellow clarified liquid, 22 kinds of intravenous drug solutions were colorless or almost colorless clarified liquid. The combination solutions of XBJ with other 53 drugs solutions were mainly yellow or browish-yellow clarified liquid, which was also suitable for turbidity determination. The turbidity change of the solution can avoid the possible influence of color on turbidity, and physical incompatibility was defined as ≥0.5 NTU change in turbidity compared to 0 h [13, 14]. From above tests we found that XBJ was incompatible with Iprrazole sodium and Rabeprazole sodium.

Part 4 of the Ch.P recommends that injectable solutions be analyzed using a light obscuration particle count test [10]. For this study, XBJ with Danshen Chuanxiongqin injection, Dengzhanxixin injection, Guanxinning injection, Kang’ai injection, Kuhuang injection, Qingkailing injection, Shenfu injection, Xingnaojing injection, Xueshuantong for injection, Aciclovir sodium, Cefoperazone sodium and Sulbactam sodium (1:1), Ceftriaxone sodium, Cefuroxime sodium, Composite Potassium Hydrogen Phosphate, Ganciclovir sodium, Ilaprazole sodium, Rabeprazole sodium solutions result in particles exceed the limitation of the Ch.P immediately after mixing and also after 4 hours. The particles in XBJ + Tanreqing injection and XBJ + Cefazolin sodium solutions exceed the limitation of the Ch.P at the time of 4 h, indicating that XBJ was incompatible with above 19 drugs. Interestingly, Tyndall Beam presented in the combination of XBJ + Acyclovir sodium, XBJ + Ceftriaxone sodium, XBJ + Cefuroxime sodium, XBJ + Iprazole sodium and XBJ + Rabeprazole sodium groups, indicated that exceed the limitation of particles might produce Tyndall Beam. During the test, XBJ and select drug were slowly diluted in NS or D5W respectively, mixed slowly along the bottle wall to avoid air bubbles affecting the count of particles. If bubbles are formed, solutions were recommended to stand briefly or subjected to ultrasonic defoaming for 1 min. It was found that XBJ diluted with NS or D5W with concentration ≥0.5 ml/ml might result in exceeding the limitation of particles and produce Tyndall Beam. Thus, it was recommended to use XBJ concentration ≤0.33 ml/ml clinically. Our pre-experiment found that particles counts were not suitable for the quality control of oily Chinese medicine injectable emulsion such as Brucea Javanica oil emulsion injection, Elemene injectable emulsion and Zedoray Turmeric oil injection, which were suitable for "Determination of particle size and particle size distribution method". The further studies will conduct in next stage.

Studies reported that pH changes of intravenous drug solution within the range of 0.2 to 1.0, the solution was stable and compatible [19–22]. Dotson et al. defined physical incompatibility as a pH value of no more than 10% change from baseline (0 hour) after 48 hours [15]. For our pH measurements (Table 4), all solutions were within the range of 4.0 to 10.0. Compared with 0h, if the pH value of the solution changes by 10%, the pH result will change within 0.4~1.0 accordingly. We intentionally to select the stricter pH changes defined as <10% to confer compatibility in our study, and all solutions had pH change <10% over the time of test. Compare to visual measurement, using a colorimeter to determine the chromacity value of the solutions have advantages in accurately and quantitatively. However, turbid solutions, viscosity solutions or fluorescent solutions will affect the transmission of the inspection light, they are not suitable for chromacity value measurement. Binary combinations of XBJ with selected 53 intravenous drug solutions are clear, non-viscous, non-fluorescent liquid, so it is suitable for chromacity value determination. From results in Table 4, chromacity value of all solutions were ≥700 and had no change in color visually with 4 h. We inferred that the chromacity value changes are less than 200, two drugs were considered physically compatible. Based on previously published literatures, colorless, almost colorless or lightly colored drug solutions were considered compatible if A_420 nm_ <0.0400 [16, 19]. Generally, the absorbance of test solution falls between 0.3 and 0.7, indicating a small margin of measurement error. Binary combinations of XBJ with 53 intravenous drug solutions were primarily yellow or brown-yellow in appearance. The range of A_420 nm_ varied from 2.8000 to 4.1000, resulting in a significant error. The change in A_420 nm_ exceeded 0.0400 (Table 4). The results indicated that criterion of A_420 nm_ was not suitable for physically compatibility of our study. The light transmittance of water for injection at A_550 nm_ is 100% and the absorbance is 0. Studies reported that drug solutions were considered compatible if A_550 nm_ <0.0100 [16, 19]. 21 drugs were incompatible with XBJ during the test.

Due to the lack knowledge on drug incompatibility issues and ways on how to avoid them, incompatibility is often under-recognised by health care practitioners. Incorrect processing with incompatibility events will bring consequences for workload, infection and cost [23]. How to handle y-site the situation need more studies to explore and verify. However, when co-administration is inevitable, flushing or filter is needed [24]. Chinese medicine injections primarily consist of water extracts from complex Chinese herbs. During storage and application, the insoluble particles can increase, which may pose potential risks. To ensure the safety of these injections, it is advisable to use a disposable precision filter infusion set with a 5.0 μm diameter before intravenous infusion. Additionally, proper storage conditions should be maintained to prevent contamination by microorganisms, ensuring the physical, chemical, and pharmacodynamic stability of the drugs. However, chemical and pharmacodynamic measurements are time-consuming and limited by instruments and experimental conditions, which do not meet the requirements for obtaining rapid results. Therefore, the evaluation of Chinese medicine injections should primarily focus on physical compatibility criteria, including visual inspection, Tyndall beam, turbidity, particles, pH, chromacity, and absorption at A550 nm. Nevertheless, considering chemical compatibility can provide further insights into the quality of the solutions.

## 5. Conclusions

The study clearly illustrated the physical compatibility of combinations of XBJ with selected 53 intravenous drugs. Of the 53 tested drugs, our findings demonstrated that XBJ was physically incompatible with 27 intravenous drugs, including 13 Chinese medicine injections: Danshen Chuanxiongqin injection, Dengzhanxixin injection, Extract of Ginkgo Biloba Leaves, Guanxinning injection, Kang’ai injection, Kuhuang injection, Qingkailing injection, Shenfu injection, Tanreqing injection, Xiangdan injection, Xingnaojing injection, Yinxingneizhi injection, Xueshuantong for injection,14 chemical drugs: Aciclovir sodium, Cefazolin sodium, Cefuroxime sodium, Ceftriaxone sodium, Cefoperazone sodium and Sulbactam sodium (1:1), Composite Potassium Hydrogen Phosphate injection, Doxofylline, Ganciclovir sodium, Ilaprazole sodium, Lansoprazole sodium, Magnesium Sulfate, Omeprazole sodium, Pantoprazole sodium and Rabeprazole sodium (Table 5). A total of 26 drugs were compatible with XBJ. XBJ should not be simultaneously co-administered with above 27 drugs through the Y tube. If coadministration is necessary, it is recommended to flush the infusion tube with an appropriate amount of NS or D5W before and after infusion of XBJ. The novel findings on the physical compatibility of XBJ broadens current knowledge of the safe coadministration of intravenous drugs clinically. It provides a safer infusion in ward. Additionally, we provide scientific and feasible methods for compatibility testing of Chinese herbal injection with other intravenous drugs for the quality control of infusion. These findings have significant implications for clinical practice.

**Table 5:** Description of XBJ physical incompatibilities with 27 drugs.

Drug	Time after mixing with XBJ			
	Immediately	1 h	2 h	4 h
Aciclovir Sodium	a, b	a, b, c	a, b, c	a, b, c, d
Cefazolin Sodium	-	-	-	b
Cefoperazone Sodium and Sulbactam Sodium (1∶1)	b	b	b	b
Ceftriaxone Sodium	a, b	a, b, d	a, b, d	a, b, c, d
Cefuroxime Sodium	b	b, d	a, b, c, d	a, b, c, d
Composite Potassium Hydrogen Phosphate	b	b	b	b
Danshen Chuanxiongqin injection	b	b	b, d	b, c, d
Dengzhanxixin injection	b	b	b	b, c
Doxofylline	-	-	-	d
Extract of Ginkgo Biloba Leaves	-	-	-	d
Ganciclovir sodium	-	b, c, d	b, c, d	b, c, d
Guanxinning injection	b	b, d	b, d	b, d
Ilaprazole Sodium	a, b, c	a, b, c, d, e	a, b, c, d, e	a, b, c, d, e
Kang’ai injection	b	b, d	b, d	b, c, d
Kuhuang injection	b	b, d	b, d	b, d
Lansoprazole	-	d	d	d
Magnesium Sulfate	-	-	-	d
Omeprazole Sodium	-	d	d	d
Pantoprazole Sodium	-	d	d	d
Qingkailing injection	b	b	b	b, d
Rabeprazole Sodium	a, b, c	a, b, c, d, e	a, b, c, d, e	a, b, c, d, e
Shenfu injection	b	b	b	b
Tanreqing injection	-	d	d	b, d
Xiangdan injection	-	-	d	d
Xingnaojing injection	-	-	b	b
Yinxingneizhi injection	-	-	-	d
Xueshuantong for injection	b	b, d	b, d	b, d

**Note:** Tyndall positive = a, ≥10 μm particles exceed 25 particles/ml = b, ≥25 μm particles exceed 3 particles/ml = c, change of A_550 nm_ ≥0.0100 = d, turbidity increased by ≥0.5NTU = e

## Supporting information

S1 Raw data (XLSX)
