# Correction: Physical compatibility of Xuebijing injection with 53 intravenous drugs during simulated Y-site administration

**DOI:** 10.1371/journal.pone.0307827

**Published:** 2024-07-22

**Authors:** Tong Tong, Peifang Li, Haiwen Ding, Ying Huang, Sheng Liu

In the 2.11 Definition of compatibility subsection of the Materials and methods, there is an error in the second sentence of the first paragraph. The correct sentence is: or absorption changes by A420 nm <0.0400 or A550 nm <0.0100 compared to 0 h.
