# Supplementary material for: Physical compatibility of Xuebijing injection with 53 intravenous drugs during simulated Y-site administration
Source: PLoS One. 2024 Mar 22;19(3):e0299694. doi: 10.1371/journal.pone.0299694 (PMC10959390; doi:10.1371/journal.pone.0299694)
Supplement: Attachment — Submitted filename: Response to Reviewers.docx [file pone.0299694.s002.docx]

Dear Editor and Reviewers,

Thanks very much for taking your time to review this manuscript. I really appreciate all your comments and suggestions! Please find my itemized responses in below and my revisions/corrections in the re-submitted files.

Thanks again!

**Reviewers’ Comments:**

Reviewer #1: This compatibility research is important to provide practical recommendations in hospitals. As the best of our knowledge, the drugs used in the study have never been tested for compatibility, so they provide novelty value. In addition, this study perhaps will provide positive enthusiasm for research in the herbal field, because herbal medicines can be made in injection preparations that are stable and compatible with other injection preparations.
This research has used an excellent method to test the compatibility of injectable drugs. The manuscript also shows the method in detail. Improvement may be required regarding this.

**Comments:**

1. How is the final justification for incompatibility taking into account all tests. So that in the results section, readers can understand where the number 27 incompatible drugs comes from?
   It is necessary to state that "the incompatibility results of one of the tests conclude that there is incompatibility". Or it would be clearer if the justification were shown in the picture. As an illustration, perhaps you can see figure 1 from this publication Compatibility of acetaminophen with central nervous system medications during simulated Y-site injection. Anaesthesiology Intensive Therapy. 2020; 52(1): 23-27. doi:10.5114/ait.2020.92684.

**Response**

Thank you for valuable review and comments on our manuscript. You are right. We have combined these justification of physical incompatibilities into a figure from the publication you suggested us.

2. The results section will be easier to read if it is displayed in a table with columns of results per test.

**Response**

Thank you for your professional suggestion. We have added all tables under results section per test and made some simplifications according to your valuable comments.

3. In the discussion section it may be necessary to briefly discuss how to manage incompatible drugs for practical purposes. Please refer to the results of this publication "Management of Y-Site Incompatibility of Intravenous Medication: A Scoping Review." Indonesian Journal of Pharmacy/Indonesian Pharmacy Magazine 33.3 (2022).

**Response**

Thank you for valuable review and professional suggestion to our manuscript. We have added discussion on how to manage incompatible drugs for practical purposes in the discussion section. We also carefully studied two literatures that you recommended to us at the same time.

Reviewer #2: Overall, the manuscript presents interesting findings, but the data is challenging to interpret. The data needs to be in a reader-friendly format. Easy to review, compare, and reference.

**Comments:**

1. Page 4-5. 2.4 . Tyndall Beam Assessment Lines 76-78
   Grammatical errors – Please revise - the sentence is confusing as written.
   Page 5. 2.5. Turbidity Line 82
   Grammatical errors – Please revise - the sentence is confusing as written.
   Page 5. 2.6. Particle Line 89
   Grammatical errors – Please revise - the sentence is confusing as written. When you say “three times” it is unclear what you are describing. It is an incomplete thought, are you saying you tested the same sample three times or you tested three samples?
   Grammar – mixing present and past tense

**Response**

Thank you for your suggestion. We have refined and modified the entire grammar and sentences in detail.

2. Page 10 - Lines 181-182. A confusing explanation for the change in pH. A 10% change in the pH of a solution of 4.0 is 0.4 and for 10.0 is 1.0. The authors need to clarify their statements regarding pH measurements and the 10% cut-off. “When the pH of a solution change is 0.4 to 1.0 it is not 10% unless you are specifically referencing a change in pH of 4.0 to 4.4.

**Response**

We are grateful for the suggestion. To be more clear and in accordance with your concerns, we have modified the description in manuscript as follows: “all solutions were within the range of 4.0 to 10.0. Compared with 0h, if the pH value of the solution changes by 10%, the pH result will change within 0.4~1.0 accordingly”

3. Tables are needed to understand the results for the different admixtures. Readers appreciate tables conveying the key findings.

**Response**

Thank you for your valuable comments on our paper. Your suggestions are very good. We have considered this issue before, but because the simplified table may damage the integrity of this article, we did not make major adjustments. However, we have made some simplifications according to your valuable comments, hoping to make it easier for readers to understand. I am very eager for my manuscript to be accepted by your journal.
